# Cerebrospinal Fluid Amyloid and Tau Biomarker Changes Across the Alzheimer Disease Clinical Spectrum

**DOI:** 10.1001/jamanetworkopen.2025.19919

**Published:** 2025-07-10

**Authors:** Diederick M. de Leeuw, Calvin Trieu, Ellen M. Vromen, Elena R. Blujdea, Inge M. W. Verberk, Flora H. Duits, Charlotte E. Teunissen, Yolande A. L. Pijnenburg, Wiesje M. van der Flier, Argonde C. van Harten, Pieter Jelle Visser, Betty M. Tijms

**Affiliations:** 1 Alzheimer Center Amsterdam, Neurology, Vrije Universiteit Amsterdam, Amsterdam University Medical Center, Location VUmc, Amsterdam, the Netherlands; 2 Amsterdam Neuroscience, Neurodegeneration, Amsterdam, the Netherlands; 3 Department of Clinical Chemistry, Neurochemistry Lab and Biobank, Amsterdam Neuroscience, Amsterdam University Medical Center, Location VUmc, the Netherlands; 4 Alzheimer Centrum Limburg, Maastricht University, Maastricht, Netherlands; 5 Department of Neurobiology, Care Sciences and Society, Division of Neurogeriatrics, Karolinska Institutet, Stockholm, Sweden

## Abstract

**Importance:**

The trajectories of core Alzheimer disease (AD) cerebrospinal fluid (CSF) biomarkers and the concurrent cognitive changes across the clinical spectrum remain unclear yet are important for clinical trial design.

**Objective:**

To map longitudinal CSF amyloid, tau, and cognitive trajectories along the clinical spectrum of AD and amyloid-negative controls.

**Design, Setting, and Participants:**

This longitudinal cohort study included participants with a minimum of 2 CSF samples from Alzheimer Centrum Amsterdam cohorts across the AD clinical spectrum (ie, abnormal amyloid levels at first visit, in different clinical stages) and cognitively normal controls with initially normal CSF markers from November 2003 to July 2019. The maximum follow-up period was 19.5 years (median [IQR], 2 [0-3] years). Data were analyzed from March 2024 to May 2025.

**Exposures:**

AD biomarkers (ß-amyloid [Aß]1-42 to Aß1-40 ratio, total tau [t-tau], and phosphorylated tau [p-tau]) detected in serially collected CSF.

**Main Outcomes and Measures:**

CSF AD biomarkers were measured with Lumipulse G600II. Cognition was measured using the Mini-Mental State Examination (MMSE) and delayed memory recall component of the of the Rey Auditory Verbal Learning Test. Analysis was conducted using linear mixed models, including random intercepts and slopes, adjusting for age, education level, and sex. Each model included an interaction term of time and clinical stage to study stage-specific slopes. Biomarker conversion rates per clinical stage were studied by comparing biomarker status between visits.

**Results:**

The sample included 197 individuals (103 male [52.3%]), including 83 controls (mean [SD] age, 63 [8] years), 31 individuals with amyloid positivity who were cognitively unimpaired (mean [SD] age, 67 [9] years), 30 individuals with amyloid-positive mild cognitive impairment (MCI; mean [SD] age, 67 [7] years), and 53 individuals with amyloid-positive dementia (mean [SD] age, 65 [8] years). Aβ1-42/1-40 ratios decreased in controls (β [SE] = −8.55 × 10^−4^ [1.87 × 10^−4^]; *P* < .001) and the amyloid-positive cognitively unimpaired group (β [SE] = −1.05 × 10^−3^ [3.14 × 10^−4^]; *P* < .001), and remained low in amyloid-positive MCI and dementia groups. There were 10 controls (12.0%) who reached abnormal amyloid over a mean (SD) of 4.8 (3.4) years. In controls, CSF t-tau (β [SE] = 8.49 [2.55] pg/mL per year; *P* = .002) and p-tau (β [SE] = 1.36 [0.41] pg/mL per year; *P* = .001) levels increased over time, and levels also increased for those in the amyloid-positive cognitively unimpaired (t-tau: β [SE] = 17.24 [4.58] pg/mL per year; *P* < .001; p-tau: β [SE] = 3.10 [0.72] pg/mL per year; *P* < .001) and amyloid-positive MCI (t-tau: β [SE] = 30.80 [5.99] pg/mL per year; *P* < .001; p-tau: β [SE] = 4.40 [0.93] pg/mL per year; *P* < .001) groups, with t-tau increasing further in the dementia group (β [SE] = 24.97 [7.80] pg/mL per year; *P* = .002). Longitudinal increases in p-tau and t-tau were steeper in the amyloid-positive cognitively unimpaired and MCI groups than in controls, with 10 controls (12.0%) reaching abnormal p-tau and 12 controls (14.5%) reaching abnormal t-tau levels. Delayed recall declined most in the amyloid-positive cognitively unimpaired group (β [SE] = −0.31 [0.07]; *P* < .001) and was associated with CSF amyloid levels (β [SE] = 102.29 [47.30]; *P* = .03). MMSE scores declined most in individuals with amyloid-positive MCI (β [SE] = −1.25 [0.12]; P < .001) and dementia (β [SE] = −1.89 [0.13]; *P* < .001).

**Conclusions and Relevance:**

In this cohort study, CSF amyloid decreased toward abnormal levels in controls, declined further in the amyloid-positive cognitively unimpaired group , and was concurrent with decline of delayed recall; CSF amyloid stabilized in those with amyloid-positive MCI and dementia, while tau markers became increased (ie, more abnormal) in the amyloid-positive cognitively unimpaired and amyloid-positive MCI groups, suggesting that increase in CSF tau requires abnormal amyloid.

## Key Points

**Question:**

What is the natural course of cerebrospinal fluid (CSF) Alzheimer disease (AD) biomarkers across the AD continuum, and how are these associated with cognitive decline?

**Findings:**

This cohort study of 197 individuals found that CSF amyloid started to reach abnormal levels in controls (with further decreases in those with amyloid positivity who were cognitively unimpaired) and was associated with decline in delayed recall. The increase of CSF tau markers was accelerated in those with amyloid positivity who were cognitively unimpaired and those with amyloid-positive mild cognitive impairment and was associated with cognitive decline.

**Meaning:**

These findings suggest that the optimal timing for secondary intervention is before the accelerated increase of CSF tau markers in individuals with amyloid positivity without cognitive impairment.

## Teaser

This cohort study analyzes changes in cerebrospinal fluid (CSF) biomarkers and cognition among individuals in different stages of the Alzheimer disease clinical spectrum.

## Introduction

Alzheimer disease (AD) is a neurodegenerative disorder that is the cause of dementia in 60% to 70% of cases. AD is pathologically defined with the presence of amyloid plaques and tau tangles in the brain.^1^ It is hypothesized that the disease starts with abnormal aggregation of amyloid, consecutively followed by hyperphosphorylation and aggregation of tau, and eventually followed by cognitive decline years later.^2^ The presence of these pathologies can be detected in cerebrospinal fluid (CSF), and these CSF biomarkers have been incorporated in the biological definition of AD in the revised criteria published by the Alzheimer Association convened workgroup.^3,4^ More than 200 studies to date have found robust associations of CSF biomarker measurements of the main pathologies with risk of cognitive decline in the predementia stages.^5,6,7^ Therefore, the field is moving toward earlier AD disease stages to identify targets for disease modification or prevention. CSF biomarkers play an important role in these studies to measure biologically relevant end points and demonstrate treatment response.^8^ Given the increasing use of CSF markers as outcomes in clinical trials, it is important to understand their trajectories and their association with cognitive decline.

To date, 6 cohorts have measured serial CSF amyloid and tau over time and investigated the longitudinal changes in repeatedly measured CSF AD biomarkers.^9,10,11,12,13,14,15,16,17^ The results of these studies are in accordance with hypothetical models where the pathological cascade starts with the accumulation of amyloid followed by tau and cognitive decline.^2^ In addition, when individuals reach mild cognitive impairment (MCI), the buildup of tau seems to be accelerated.^10,15^ All these studies focused on the estimated change of CSF biomarkers and cognition in association with the onset of amyloid-positive dementia (ie, how many years before onset of amyloid-positive dementia does the change in CSF biomarkers start to occur). However, the temporal behavior of these CSF biomarkers per disease stage across the AD continuum remains understudied. A better understanding of CSF AD biomarker trajectories is paramount for clinical trial design to determine the study population, optimal time of intervention, and the treatment response.

The objective of the present study was to investigate changes in CSF biomarkers in a cohort of older adults with normal biomarkers and cognition and in individuals across the AD continuum. We assessed changes over a period of up to 14 years in AD hallmark CSF measures: ratio of ß-amyloid (Aß) 1-42 to Aß1-40, total tau (t-tau), and phosphorylated tau-181 (p-tau). Finally, we studied to what extent biomarker changes were concurrent with decline in memory functioning over time across the AD spectrum.

## Methods

### Participants

This cohort study followed the Strengthening the Reporting of Observational Studies in Epidemiology (STROBE) reporting guideline. We included individuals who had repeated CSF sampling available and who were initially cognitively unimpaired amyloid-negative controls or had positive amyloid markers with either intact cognition, MCI, or dementia, from studies performed at the Alzheimer Center Amsterdam: Amsterdam Dementia Cohort (ADC), the Subjective Cognitive Impairment Cohort, and the European Medical Information Framework for Alzheimer’s Disease PreclinAD study cohort. Briefly, participants of the ADC receive regular care within the memory clinic of the Alzheimer Center Amsterdam and are re-evaluated on an annual basis.^18^ At first visit, these individuals undergo extensive dementia screening including neuropsychological testing, lumbar puncture, and brain imaging. The results are discussed in multidisciplinary meetings to reach a consensus diagnosis according to diagnostic and research guidelines of all major neurodegenerative diseases.^19,20,21,22,23,24,25,26,27,28,29,30^ ADC participants included in the present study were examined between November 2003 and July 2019. The Subjective Cognitive Impairment Cohort study includes a subgroup of participants from the ADC who initially present with subjective cognitive decline.^31^ These individuals are followed yearly with clinical assessment, neuropsychological examination, and laboratory analysis (plasma and CSF) to identify progression from subjective cognitive decline to MCI and dementia. Lastly, the European Medical Information Framework for Alzheimer’s Disease PreclinAD study enrolled cognitively normal monozygotic twins aged 60 years and older from the Netherlands Twins Register to capture the earliest changes of Alzheimer disease in cognitively normal adults.^32^ For this study, we included only unrelated individuals. This study did not require informed consent because all included participants had previously provided written informed consent to use their clinical data and biospecimens. Local institutional review boards approved these studies.

### Cognitive Markers

All participants in the different cohorts underwent neuropsychological testing, including the Mini-Mental State Examination (MMSE) and the delayed recall component of the Rey Auditory Verbal Learning Test. A total of 946 scores were available for both cognitive tests, with all participants having a minimum of 1 follow-up test score, with a median of 4 time points available per participant (MMSE: range, 1-13 time points; delayed recall: range, 1-11 time points). These scores were collected over a median (IQR) follow-up period of 2 (0-2) years with maximum follow-up time ranging from 6.4 years in dementia up to 19.5 years in controls.

### CSF Analyses

CSF samples were obtained by lumbar puncture using a 25-gauge needle and syringe between the L3 and L4, L4 and L5, or L5 and S1 intervertebral space and collected in polypropylene tubes and processed and stored at −80 °C as previously described.^18^ Included participants were required to have at least 2 CSF samples.

AD biomarkers Aß-42, Aß1-40, p-tau-181, and t-tau were then measured using the Lumipulse G600II–I instrument (Fujirebio Diagnostics) in accordance with the manufacturer’s instructions. Biomarker cutoff values for all these AD biomarkers measured with the Lumipulse were previously determined based on optimizing the Youden index in receiver operating curve analyses with amyloid positron emission tomography result as proxy for the accepted standard. Amyloid positivity was defined as Aß-42/Aß1-40 ratio levels less than the predetermined cutoff value at 0.071,^33^ while abnormal tau values were determined at cutoffs 61.5 pg/mL for p-tau and 463 pg/mL for t-tau (unpublished cutoffs which were determined in the same cohort against amyloid positron emission tomography as Willemse et al^33^). See eTable 1 in Supplement 1 for concordance with historical measures of amyloid and tau with the Innotest assay.

### Statistical Analysis

We first compared groups (ie, controls, amyloid-positive cognitively unimpaired, amyloid-positive MCI, and amyloid-positive dementia) on clinical characteristics with analysis of variance for continuous variables with a normal distribution, a Kruskal-Wallis test for variables with a skewed distribution, and a χ^2^ test for categorical variables. Next, we tested changes over time with linear mixed models in each CSF biomarker and cognitive marker (ie, MMSE and delayed recall) as an outcome measure with time as a main effect and an interaction term of time × diagnostic group to test if slopes differed between the groups. We modeled random intercepts and slopes and corrected for sex and age and additionally corrected for education level for the models with cognitive markers as outcome. The significance level for all models was set at a 2-tailed *P* < .05. Furthermore, we also used linear mixed models to examine whether CSF biomarker trajectories were associated with cognitive decline over time. In these models, longitudinal cognitive test scores were entered as the dependent variable, and longitudinal CSF biomarker levels were included as exposures. For significant associations, we repeated analyses to assess to what extent biomarker intercepts and/or slopes contributed to associations with cognitive outcomes by first estimating participant-level biomarker estimates and slopes from a linear mixed model with CSF marker as the outcome, and time as the exposure with random slopes and intercepts. Next, these participant-level intercepts and slopes were entered into a second model with cognitive measures as the outcome and modeling interaction effect of time × biomarker slope and intercept as a covariate, adjusting for age, sex, and educational level. The Emmeans package (version 1.8.6) was used to model the stage specific parameters, which were used to report the outcomes per clinical group and their respective differences. Finally, we assessed progression rates by assessing biomarker status from each follow-up visit to the baseline visit for all participants, which allowed us to quantify the proportion of individuals who transitioned from normal to abnormal biomarker status over time. Additionally, we computed the average time to progression by determining the interval between baseline visits and the first visit where abnormal biomarker levels were observed. All analyses were performed with R version 4.1.1 (2021-08-10)–Kick Things (R Project for Statistical Computing) from March 2024 to May 2025.

## Results

### Sample Characteristics at First Visit

In total, 197 individuals (103 male [52.3%]) had at least 2 CSF samples available over a mean (SD) follow-up time of 2 (1) years (465 CSF samples in total), including 31 individuals in the amyloid-positive cognitively unimpaired group, 30 in the amyloid-positive MCI group, and 53 in the amyloid-positive dementia group (Table 1). Some individuals had 3 (35 participants [17.8%]) or 4 samples (24 participants [12.2%]), with follow-up time ranging from up to 5 years in amyloid-positive dementia (median [IQR] time between CSF measurements, 1 [0-1] years) and up to 14 years in controls (median [IQR] time between CSF measurements, 2 [0-4] years) (Table 1). Compared with controls (mean [SD] age, 63 [8] years), AD groups were older (amyloid-positive cognitively unimpaired: mean [SD] age, 67 [9] years; amyloid-positive MCI: mean [SD] age, 67 [7] years; amyloid-positive dementia: mean [SD] age, 65 [8] years; *P* = .03) and were more likely to carry an *APOE4* allele (control: 27 individuals [32.5%]; amyloid-positive cognitively unimpaired: 21 individuals [67.7%]; amyloid-positive MCI: 23 individuals [76.7%]; amyloid-positive dementia: 35 individuals [66.0%]; *P* < .001). Within AD, individuals in the dementia stage had the lowest median (IQR) MMSE scores (23 [20-26]) and the lowest median (IQR) number of repeated MMSE tests (3 [2-5] tests). In addition, both amyloid-positive MCI and dementia groups had the lowest median (IQR) rates of repeated delayed recall scores (amyloid-positive MCI: 4 [3-5] scores; amyloid-positive dementia: 2 [1-3] scores) and their mean (SD) delayed recall scores were similarly low (amyloid-positive MCI: 3 [2]; amyloid-positive dementia: 3 [3]). Sex did not differ between groups. By definition, AD groups had lower Aß1-42/Aß1-40 ratios and higher t-tau and p-tau CSF levels at baseline compared with controls. Furthermore, the median (IQR) follow-up time for individuals in the dementia group (1 [0-1] years) was shorter compared with other groups (amyloid-positive cognitively unimpaired: 2 [0-4] years; *P* < .001; controls: 2 [0-4] years; *P* < .001), and the median (IQR) follow-up time for individuals with amyloid-positive MCI (1 [0-2] years) was shorter than controls (*P* = .04). Finally, amyloid-positive MCI and amyloid-positive dementia had fewer repeated samples (4 participants [13.3%] and 4 participants [7.5%], respectively, had more than 2 repeated samples; *P* < .001 ) compared with controls and the amyloid-positive cognitively unimpaired group (39 participants [47.0%] and 12 participants [38.7%], respectively, had more than 2 repeated samples; *P* = .01).

**Table 1.** Demographics of Study Population

Characteristic	Participants, No. (%) (N = 197)				*P* value
	Controls (n = 83)	Amyloid-positive cognitively unimpaired (n = 31)	Amyloid-positive mild cognitive impairment (n =30)	Amyloid-positive dementia (n = 53)	
Sex					
Male	46 (55.4)	11 (35.5)	19 (63.3)	27 (50.9)	.15
Female	37 (44.6)	20 (64.5)	11 (36.7)	26 (49.1)	
*APOE4* carrier	27 (32.5)	21 (67.7)	23 (76.7)	35 (66.0)	<.001^a^^,^^b^^,^^c^
Age, mean (SD), y	63 (8)	67 (9)	67 (7)	65 (8)	.03^b^
Time between cerebrospinal fluid measurements, median (IQR), y	2 (0-4)	2 (0-4)	1 (0-2)	1 (0-1)	<.001^b^^,^^c^^,^^d^
No. of repeated samples					
2	44 (53.0)	19 (61.3)	26 (86.7)	49 (92.5)	<.001^b^^,^^c^^,^^d^
3	20 (24.1)	7 (22.6)	4 (13.3)	4 (7.5)	
4	19 (22.9)	5 (16.1)	0	0	
MMSE score, median (IQR)	29 (28-30)	29 (28-30)	26 (24-28)	23 (20-26)	<.001^b^^,^^c^^,^^d^^,^^e^^,^^f^
No. of repeated MMSEs, median (IQR)	4 (4-7)	4 (4-6)	5 (4-6)	3 (2-5)	.008^c^^,^^f^
Delayed recall score, mean (SD)	9 (3)	8 (2)	3 (2)	3 (3)	<.001^a^^,^^b^^,^^c^^,^^d^^,^^e^
No. of repeated delayed memory recall scores, median (IQR)	4 (4-6)	4 (4-5)	4 (3-5)	2 (1-3)	<.001^c^^,^^d^^,^^f^
Plasma t-tau, median (IQR), pg/mL	319.0 (253.9-421.2)	466.5 (393.5-666.2)	714.0 (566.5-868.5)	750.0 (658.2-986.5)	<.001^a^^,^^b^^,^^c^^,^^d^^,^^e^
Plasma p-tau, median (IQR), pg/mL	35.9 (28.1-48.5)	74.1 (49.0-104.8)	110.9 (88.0-144.6)	115.6 (97.4-160.6)	<.001^a^^,^^b^^,^^c^^,^^d^^,^^e^

Abbreviations: MMSE, Mini-Mental State Examination; p-tau, phosphorylated tau; t-tau, total tau.

^a^ Controls vs amyloid-positive cognitively unimpaired.

^b^ Controls vs amyloid-positive mild cognitive impairment.

^c^ Controls vs amyloid-positive dementia.

^d^ Amyloid-positive cognitively unimpaired vs amyloid-positive dementia.

^e^ Amyloid-positive cognitively unimpaired vs amyloid-positive mild cognitive impairment.

^f^ Amyloid-positive mild cognitive impairment vs amyloid-positive dementia.

### Changes in Memory Function

**Figure 1.**
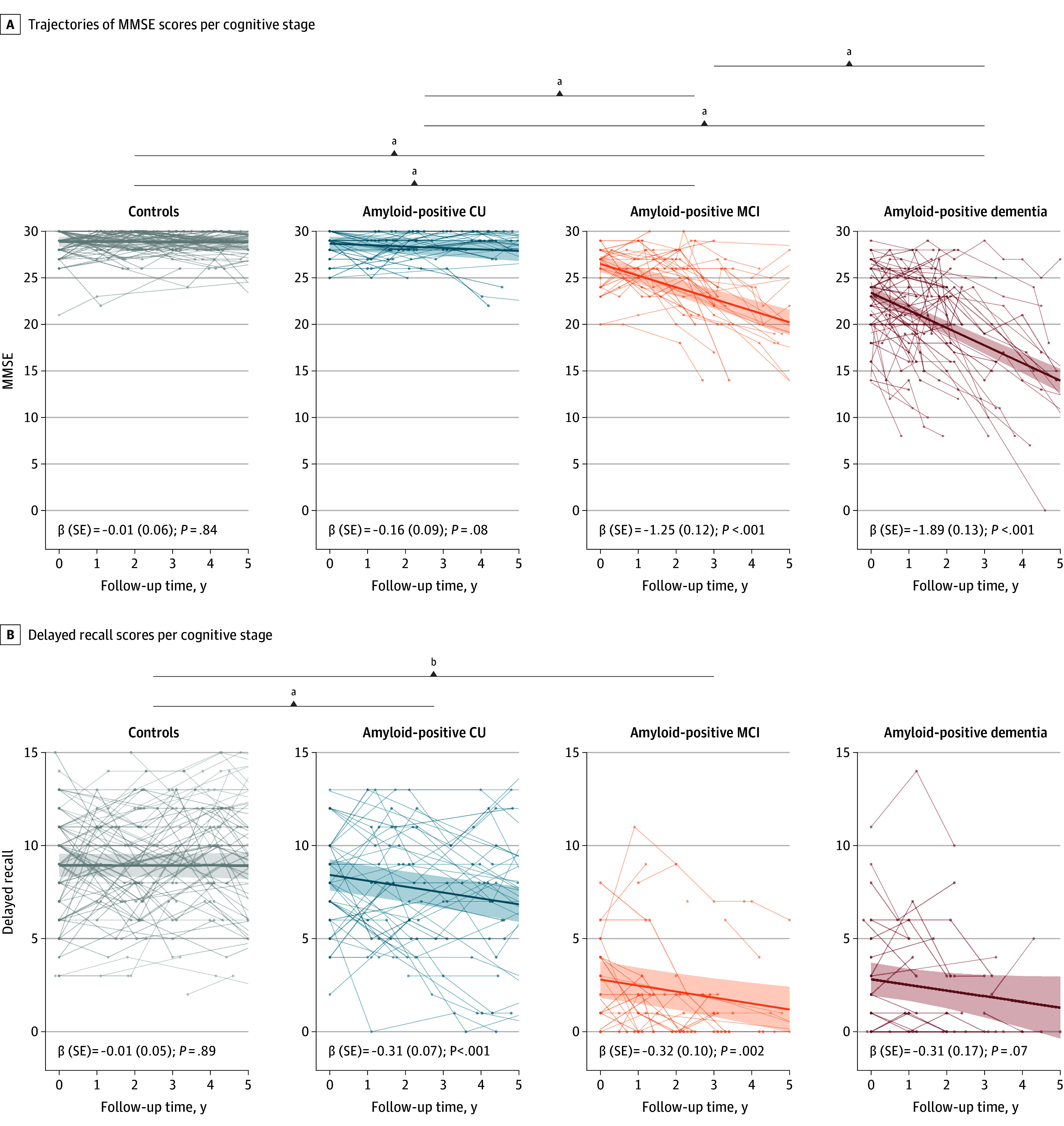
Trajectories in Cognition Across the Alzheimer Disease Spectrum A, Trajectories of Mini-Mental State Examination (MMSE) scores per cognitive stage. B, Delayed recall scores per cognitive stage. The thick line represents the slope, while the shaded area reflects the 95% CI. Lines with interconnected dots represent individual trajectories. Statistically significant differences in slopes are indicated. CU indicates cognitively unimpaired; MCI, mild cognitive impairment. ^a^*P* < .001. ^b^*P* = .005.

We next studied per-group changes over time in MMSE as a proxy for cognitive functioning, as well as the delayed memory recall test as a more sensitive measure to decline in the early stages (ie, in the amyloid-positive cognitively unimpaired group). MMSE scores did not change over time in the control and the amyloid-positive cognitively unimpaired group (Figure 1), but did decline in the amyloid-positive MCI group (β [SE] = −1.25 [0.12]; *P* < .001) and amyloid-positive dementia group (β [SE] = −1.89 [0.13]; *P* < .001) (Figure 1). Scores on the delayed recall test declined over time in the amyloid-positive cognitively unimpaired (β [SE] = −0.31 [0.07]; *P* < .001) and amyloid-positive MCI (β [SE] = −0.32 [0.10]; *P* = .002) groups; compared with controls (β [SE] = −0.01 [0.05]), the decline was significantly steeper for both the amyloid-positive cognitively unimpaired (*P* *<* .001) and amyloid positive MCI groups (*P* = .002). Decrease over time in delayed memory scores was less steep in the amyloid-positive MCI and amyloid-positive dementia groups because these scores were close to bottom level.

### Changes Over Time in CSF Markers

**Figure 2.**
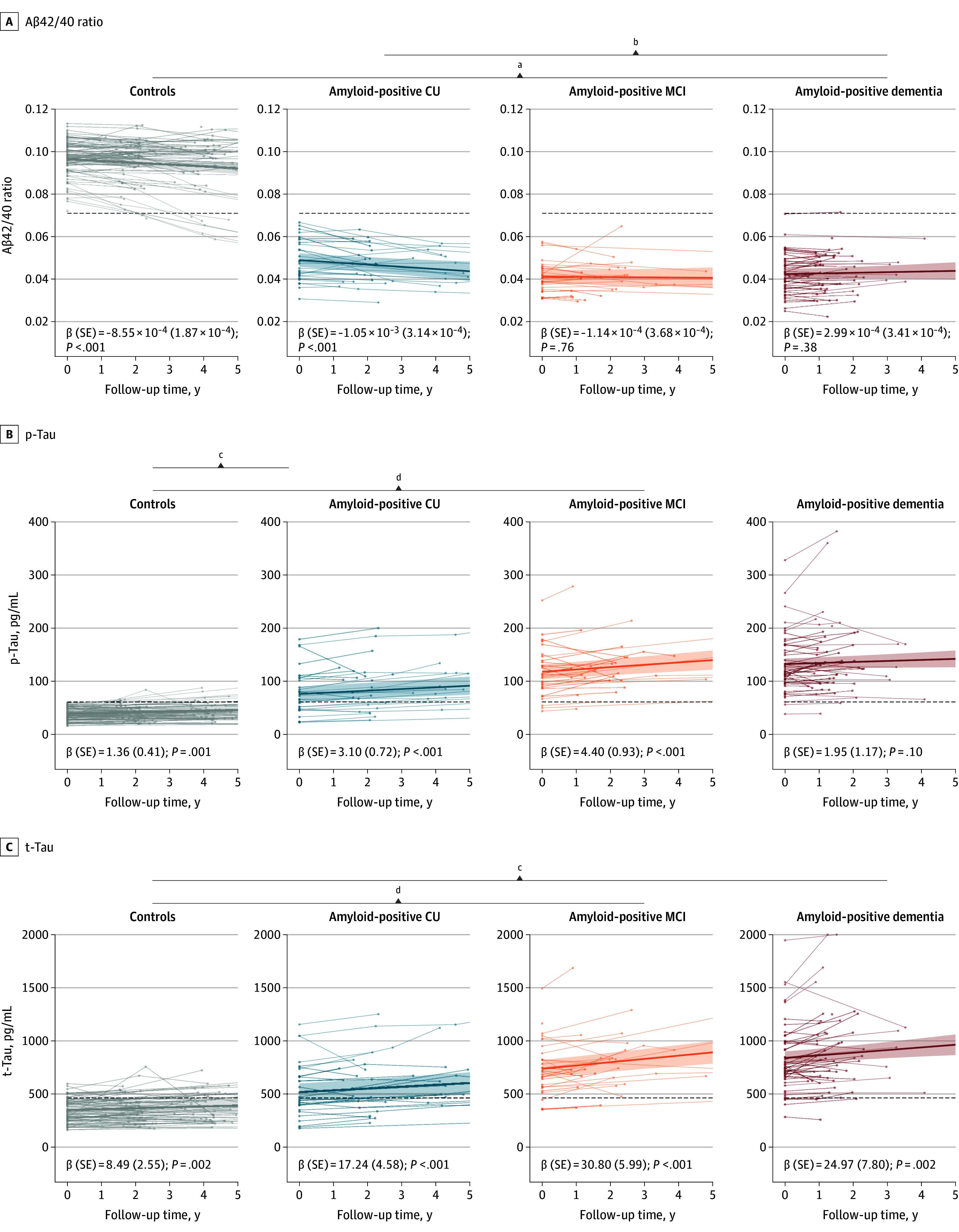
Cerebrospinal Fluid Biomarker Trajectories Across the Cognitive Spectrum ^a^*P* = .02. ^b^*P* < .001. ^c^*P* = .003. ^d^*P* = .04.

We then studied how amyloid and tau markers changed over time in all groups (Figure 2 and eTable 2 in Supplement 1). CSF Aß1-42/Aß1-40 ratio decreased in controls (ie, became more abnormal) over time (β [SE] = −8.55 × 10^−4^ [1.87 × 10^−4^]; *P* < .001), and during a mean (SD) of 4.8 (3.4) years, 10 of 83 individuals (12.0%) reached abnormal values (Table 2). In the amyloid-positive cognitively unimpaired group, CSF Aß1-42/Aß1-40 ratios had a similar decrease (β [SE] = −1.05 × 10^−3^ [3.14 × 10^−4^]; *P* < .001) compared with the decrease in the control group (*P* = .60). The CSF Aß1-42/Aß1-40 ratio did not change any further in amyloid-positive MCI (β [SE] = −1.14 × 10^−4^ [3.68× 10^−4^]; *P* = .76) and amyloid-positive dementia (β [SE] = 2.99 × 10^−4^ [3.41× 10^−4^]; *P* = .38 ) groups. The slopes of controls (*P* = .003) and the amyloid-positive cognitively unimpaired group (*P* = .004) were significantly different from the slope of the amyloid-positive dementia group.

**Table 2.** Change Over Time in Amyloid, P-Tau, and T-Tau Status in All Groups

Change in biomarker status from first visit to last visit	Participants, No. (%) (N = 197)			
	Control (n = 83)	Amyloid-positive cognitively unimpaired (n = 31)	Amyloid-positive mild cognitive impairment (n = 30)	Amyloid-positive dementia (n = 53)
Amyloid				
Normal to normal	73 (87.9)	0	0	0
Normal to abnormal	10 (12.0)	0	0	0
Abnormal to normal	0	0	0	1 (1.9)
Abnormal to abnormal	0	31 (100)	30 (100)	52 (98.1)
t-Tau				
Normal to normal	61 (73.5)	10 (32.3)	0	0
Normal to abnormal	12 (14.5)	5 (16.1)	1 (3.3)	0
Abnormal to normal	2 (2.4)	0 (0)	0	0
Abnormal to abnormal	8 (9.6)	16 (51.6)	29 (96.7)	53 (100)
p-Tau				
Normal to normal	73 (88.0)	0	0	0
Normal to abnormal	10 (12.0)	3 (9.7)	2 (6.7)	0
Abnormal to normal	0	1 (3.2)	0	0
Abnormal to abnormal	0	27 (87.1)	28 (93.3)	53 (100)

Abbreviations: p-tau, phosphorylated tau; t-tau, total tau.

When analyzing the slopes for CSF, t-tau, and p-tau over time, we observed that in controls, both t-tau (β [SE] = 8.49 [2.55] pg/mL per year; *P* = .002) and p-tau levels (β [SE] = 1.36 [0.41] pg/mL per year; *P* = .001) increased over time; there were 10 controls (12.0%) who developed abnormal p-tau and 12 controls (14.5%) who developed abnormal t-tau levels over an mean (SD) of 4.5 (2.4) years. There were increases in p-tau and t-tau in both the amyloid-positive cognitively unimpaired (t-tau: β [SE] = 17.24 [4.58] pg/mL per year; *P* < .001; p-tau: β [SE] = 3.10 [0.72] pg/mL per year; *P* < .001) and amyloid-positive MCI (t-tau: β [SE] = 30.80 [5.99] pg/mL per year; *P* < .001; p-tau: β [SE] = 4.40 [0.93] pg/mL per year; *P* < .001) groups. In the amyloid-positive dementia group, CSF t-tau levels kept increasing (β [SE] = 24.97 [7.80] pg/mL per year; *P* = .002), but this was not observed for p-tau (β [SE] = 1.95 [1.17] pg/mL; *P* = .10). Compared with controls, CSF p-tau increases were steeper in the amyloid-positive cognitively unimpaired (*P* = .04) and amyloid-positive MCI (*P* = .003) groups, while CSF t-tau increases were steeper in the amyloid-positive MCI (*P* < .001) and the amyloid-positive dementia groups (*P* = .046) compared with controls.

Finally, we studied if changes over time in CSF biomarkers were associated with changes over time in memory scores (ie, MMSE and delayed recall) (eTable 3 in Supplement 1). In these analyses, we excluded groups and tests that did not change over time (ie, controls because their MMSE and delayed recall did not change over time and the MMSE for the amyloid-positive cognitively unimpaired group). In the amyloid-positive cognitively unimpaired group, we observed that that decreases in Aß1-42/Aß1-40 ratio levels were associated with decline on the delayed recall test (β [SE] = 102.29 [47.30]; *P* = .03); there was no association for controls (β [SE] = −98.00 [52.10]; *P* *=* .06). Repeating the analysis with the slope of Aß1-42/Aß1-40 ratio included in the model, we observed that the association was solely due to amyloid positivity rather than amyloid trajectories (β [SE] = −32.70 [52.70]; *P* = .53) (eTable 4 in Supplement 1). There was no association of changes in t-tau or p-tau levels over time with decline in delayed memory in the amyloid-positive cognitively unimpaired group. In the amyloid-positive MCI group, higher p-tau and t-tau levels were associated with lower MMSE scores over time (p-tau: β [SE] = −3.46 × 10^−2^ [1.02 × 10^−2^]; *P* < .001; t-tau: β [SE] = −7.13 × 10^−3^ [1.74 × 10^−3^]; *P* < .001), and for t-tau, with steeper decline on the delayed recall (β [SE] = −4.25 × 10^−3^; [2.03× 10^−3^]; *P* = .04). Repeating these analyses with the slopes of p-tau and t-tau as an exposure, we found that the slopes of p-tau and t-tau contributed to these associations in addition to p-tau and t-tau intercepts for the MMSE (p-tau: *P* for slope = .02; t-tau: *P* for slope = .03), while the intercepts of p-tau contributed to the association with delayed recall (*P* for slope = .31). In the amyloid-positive dementia group, steeper changes in p-tau and t-tau levels over time were associated with steeper decline on the delayed recall test (p-tau: β [SE] = −3.46 × 10^−2^ [1.02 × 10^−2^]; *P* < .001; t-tau: β [SE] = −7.13 × 10^−3^ [1.74 × 10^−3^]; *P* <.001) (eTable 3 in Supplement 1). Neither the slopes nor intercepts of p-tau (*P* for intercept = .94; *P* for slope = .68) or t-tau markers (*P* for intercept = .58; *P* for slope = .92) contributed to these associations.

## Discussion

The aim of this cohort study was to investigate the dynamics of CSF amyloid and tau markers and cognitive performance over time in amyloid-negative cognitively unimpaired controls and across the clinical spectrum of AD. Changes in amyloid markers and status were most apparent in controls, with 12% progressing from normal to abnormal amyloid within 5 years, and these changes were accompanied by increases in t-tau and p-tau CSF levels. Furthermore, in individuals with abnormal amyloid at baseline, the largest changes over time in CSF t-tau and p-tau concentrations were observed in preclinical and amyloid-positive MCI stages. Moreover, delayed recall already started to decline in the amyloid-positive cognitively unimpaired group, and this decline was associated with amyloid abnormality. Our findings fit with the hypothesis that abnormal amyloid is a prerequisite for cognitive changes in amyloid-positive cognitively unimpaired individuals, and that this accelerates pathological alterations in CSF tau markers over time.

An annual decline in CSF Aß1-42/1-40 ratio was already observed in controls, and our findings show that the ratio further decreased in the amyloid-positive cognitively unimpaired group, where amyloid was already below the clinical thresholds for abnormality. In amyloid-positive MCI and amyloid-positive dementia, CSF Aß1-42/1-40 ratios seem to have reached floor levels, which is in line with previous studies^10,16,34^ and adds to the existing evidence of stabilization of CSF amyloid levels around the onset of clinical symptoms (ie, amyloid-positive MCI).^34,35,36,37,38,39^ In the amyloid-positive cognitively unimpaired group, lower levels of Aß1-42/1-40 ratios (ie, amyloid positivity) were associated with increased risk of decline on the delayed memory recall test. Recently, a debate has emerged as to whether individuals with normal cognition and abnormal amyloid can be considered to have cognitive impairment^4^ or are asymptomatic but at risk.^3^ Our results suggest that in this group, abnormal amyloid is not benign because this group also demonstrated decline in the memory domain, which is in line with earlier findings in the Alzheimer Disease Neuroimaging Initiative cohort.^40,41^ Furthermore, in our control group, we observed that 12% of controls with initial mean age of 63 years developed amyloid positivity within 5 years, which is more than twice as large as the 4.5% increase in amyloid positivity in a similar time window between the age of 65 to 70 years reported in previous large-scale cross-sectional studies.^42^ Additional analyses in the Alzheimer Disease Neuroimaging Initiative cohort indicated similar percentage of individuals developing abnormal amyloid.^42^ Possibly, this finding may be explained by the fact that in the cross-sectional studies, the percentage of individuals with normal cognition with higher age may already have progressed to MCI within 5 years; thus, the pool of individuals could change. In addition, we found that CSF p-tau and t-tau levels increased together with Aß1-42/1-40 ratios in controls. This finding implies that the onset of biomarker abnormality of amyloid and tau markers in CSF are closely tied, which we previously also observed in another independent sample.^43^ Rates of CSF t-tau and p-tau increased 2- to 3.5-fold in the amyloid-positive cognitively unimpaired and amyloid-positive MCI groups, suggesting that once amyloid is abnormal, CSF tau markers start to accelerate; this may suggest that p-tau and t-tau increases over time may reflect different aspects of AD pathophysiological processes or could even be associated with normal aging or non-AD pathology.^43,44,45,46^ Still, elevated p-tau levels in absence of abnormal amyloid may have less clinical meaning because previous studies showed that in individuals with normal cognition, abnormal amyloid (with or without abnormal CSF p-tau concentrations) is a larger predictor of clinical progression than abnormal p-tau alone.^47,48^ Moreover, in groups with abnormal amyloid markers (ie, in the amyloid-positive cognitively unimpaired and amyloid-positive MCI groups), CSF tau markers increased more steeply compared with the amyloid negative group, which is supportive of earlier studies that show amyloid accumulation precedes abnormal CSF tau levels and cognitive decline as seen in AD.^47,48,49,50,51,52,53^ Another implication of the notion that the acceleration in CSF tau levels over time in the amyloid-positive cognitively unimpaired and amyloid-positive MCI stages implies that interventions targeting amyloid in early stages may also prevent the increase of CSF tau markers. Even though this does not necessarily prevent accumulation of tau tangles, the alterations in CSF amyloid and CSF tau seem to be closely tied, which makes this finding important for clinical trials using amyloid as an outcome measure or as a target because this natural trajectory proves useful for trial design and determination of the ideal moment of intervention. Because high CSF tau has been associated with faster decline, these results would suggest individuals with amyloid positivity but no cognitive impairment and normal CSF tau levels could benefit from intervention.

### Limitations

A potential limitation of the current study is that, although we used cutoffs to determine CSF abnormality which were previously defined as having the best concordance with amyloid positron emission tomography,^54,55,56,57^ we cannot exclude the possibility that these cutoffs may still be inaccurate. Future studies should aim to keep following these individuals over time and study such findings together with pathological examinations. Furthermore, although for a memory clinic sample we included a large group of individuals with repeated CSF collection, the current sample size limits the generalizability, and future studies should focus on larger sample sizes to increase understanding of natural disease history in sporadic AD.

## Conclusions

In this cohort study of amyloid-negative cognitively unimpaired controls and individuals on the AD continuum, we analyzed the natural trajectories of CSF biomarkers of AD. We observed that CSF amyloid became decreased in controls and declined further in the amyloid-positive cognitively unimpaired group. Amyloid positivity was associated with decline of delayed recall. CSF amyloid stabilized in the amyloid-positive MCI and dementia groups. CSF tau markers became more abnormal in the amyloid-positive cognitively unimpaired and MCI groups. Based on these results, individuals with amyloid positivity but no cognitive impairment appear to be the best candidates for intervention in clinical trials because the most changes occur in this stage, such as accelerated CSF tau accumulation and cognitive decline.

## Notes

Supplement 1. **eTable 1.** Concordance of Baseline Innotest Amyloid Status and Baseline Lumipulse Aß1-42/1-40 Ratio Per Diagnostic Group **eTable 2.** Stage Specific Parameters for All CSF Biomarkers and Cognitive Tests by Estimated Marginal Means of Linear Trends (Emtrends) **eTable 3.** Stage Specific Parameters for All Associations Between Cognitive Tests and CSF Biomarkers by Estimated Marginal Means of Linear Trends (Emtrends) **eTable 4.** Associations Between Cognitive tests and CSF Biomarkers Slopes and Intercepts

Supplement 2.
Data Sharing Statement
