# Supplementary material for: Cerebrospinal Fluid Amyloid and Tau Biomarker Changes Across the Alzheimer Disease Clinical Spectrum
Source: JAMA Netw Open. 2025 Jul 10;8(7):e2519919. doi: 10.1001/jamanetworkopen.2025.19919 (PMC12246881; doi:10.1001/jamanetworkopen.2025.19919)
Supplement: Supplement 1. — eTable 1. Concordance of Baseline Innotest Amyloid Status and Baseline Lumipulse Aß1-42/1-40 Ratio Per Diagnostic Group eTable 2. Stage Specific Parameters for All CSF Biomarkers and Cognitive Tests by Estimated Marginal Means of Linear Trends (Emtrends) eTable 3. Stage Specific Parameters for All Associations Between Cognitive Tests and CSF Biomarkers by Estimated Marginal Means of Linear Trends (Emtrends) eTable 4. Associations Between Cognitive tests and CSF Biomarkers Slopes and Intercepts [file jamanetwopen-e2519919-s001.pdf]

Supplemental Online Content

de Leeuw DM, Trieu C, Vromen EM, et al. Cerebrospinal fluid amyloid and tau biomarkers trajectories changes across the Alzheimer disease clinical spectrum. *JAMA Netw. Open*. 2025;8(7):e2519919. doi:10.1001/jamanetworkopen.2025.19919

eTable 1. Concordance of Baseline Innotest Amyloid Status and Baseline Lumipulse Aß1-42/1-40 Ratio Per Diagnostic Group
eTable 2. Stage Specific Parameters for All CSF Biomarkers and Cognitive Tests by Estimated Marginal Means of Linear Trends (Emtrends)
eTable 3. Stage Specific Parameters for All Associations Between Cognitive Tests and CSF Biomarkers by Estimated Marginal Means of Linear Trends (Emtrends)
eTable 4. Associations Between Cognitive tests and CSF Biomarkers Slopes and Intercepts

This supplemental material has been provided by the authors to give readers additional information about their work.

© 2025 de Leeuw DM et al. *JAMA Network Open*.

eTable 1. Concordance of Baseline Innotest Amyloid Status and Baseline Lumipulse Aß1-42/1-40 Ratio Per Diagnostic Group

**Panel**	Controls		CU A+		MCI A+		Dementia A+	
	**Lumipulse A-**	**Lumipulse A+**	**Lumipulse A-**	**Lumipulse A+**	**Lumipulse A-**	**Lumipulse A+**	**Lumipulse A-**	**Lumipulse A+**
**Innotest A-**	79	0	0	5	0	0	0	1
**Innotest A+**	0	0	0	26	0	29	0	47
**NA***	4	0	0	0	0	1	0	5

All lumipulse samples were present. Therefore, NAs presented here are missing data for innotest.

© 2025 de Leeuw DM et al. *JAMA Network Open*.

eTable 2. Stage Specific Parameters for All CSF Biomarkers and Cognitive Tests by Estimated Marginal Means of Linear Trends (Emtrends)

**Biomarker/Cognitive marker**	**Stage**	**Emtrend estimate (SE)**	**P value**	**Stage contrast**	**Contrast estimate**	**P value**
**Aß1-42/ Aß1-40 ratio**	Controls	-8.55e-4 (1.87e-4)	**<0.001**	Controls vs CU A+	1.91e-4 (3.65e-4)	0.60
	CU A+	-1.05e-3 (3.14e-4)	**0.001**	Controls vs MCI A+	-7.42e-4 (4.13e-4)	0.07
	MCI A+	-1.14e-4 (3.68e-4)	0.76	Controls vs Dementia A+	-1.15e-3 (3.89e-4)	**0.003**
	Dementia A+	2.99e-4 (3.41e-4)	0.38	CU A+ vs MCI A+	-9.33e-4 (4.84e-4)	0.06
				CU A+ vs Dementia A+	-1.35e-3 (4.64e-4)	**0.004**
				MCI A+ vs Dementia A+	-4.13e-4 (5.02e-4)	0.41
**Biomarker/Cognitive marker**	**Stage**	**Emtrend estimate (SE)**	**P value**	**Stage contrast**	**Contrast estimate**	**P value**
**pTau**	Controls	1.36 (0.41)	**0.001**	Controls vs CU A+	-1.74 (0.83)	**0.04**
	CU A+	3.10 (0.72)	**<0.001**	Controls vs MCI A+	-3.04 (1.01)	**0.003**
	MCI A+	4.40 (0.93)	**<0.001**	Controls vs Dementia A+	-0.58 (1.24)	0.64
	Dementia A+	1.95 (1.18)	0.10	CU A+ vs MCI A+	-1.30 (1.18)	0.27
				CU A+ vs Dementia A+	1.16 (1.38)	0.40
				MCI A+ vs Dementia A+	2.46 (1.50)	0.10

© 2025 de Leeuw DM et al. *JAMA Network Open*.

**tTau**	Controls	8.49 (2.55)	**0.002**	Controls vs CU A+	-8.75 (5.24)	0.10
	CU A+	17.24 (4.58)	**<0.001**	Controls vs MCI A+	-22.31 (6.51)	**<0.001**
	MCI A+	30.80 (5.99)	**<0.001**	Controls vs Dementia A+	-16.48 (8.21)	**0.05**
	Dementia A+	24.97 (7.80)	**0.002**	CU A+ vs MCI A+	-13.56 (7.54)	0.07
				CU A+ vs Dementia A+	-7.73 (9.05)	0.40
				MCI A+ vs Dementia A+	5.84 (9.83)	0.55
**Biomarker/Cognitive marker**	**Stage**	**Emtrend estimate (SE)**	**P value**	**Stage contrast**	**Contrast estimate**	**P value**
**MMSE**	Controls	-0.01 (0.06)	0.84	Controls vs CU A+	0.14 (0.11)	0.20
	CU A+	-0.16 (0.09)	0.08	Controls vs MCI A+	1.24 (0.14)	**<0.001**
	MCI A+	-1.25 (0.12)	**<0.001**	Controls vs Dementia A+	1.87 (0.14)	**<0.001**
	Dementia A+	-1.89 (0.13)	**<0.001**	CU A+ vs MCI A+	1.10 (0.15)	**<0.001**
				CU A+ vs Dementia A+	1.73 (0.16)	**<0.001**
				MCI A+ vs Dementia A+	0.63 (0.18)	**<0.001**
**Delayed Recall**	Controls	-0.01 (0.05)	0.89	Controls vs CU A+	0.31 (0.08)	**<0.001**

© 2025 de Leeuw DM et al. *JAMA Network Open*.

	CU A+	-0.32 (0.07)	**<0.001**	Controls vs MCI A+	0.31 (0.11)	**0.005**
	MCI A+	-0.32 (0.10)	**0.002**	Controls vs Dementia A+	0.30 (0.18)	0.09
	Dementia A+	-0.31 (0.17)	0.07	CU A+ vs MCI A+	0.01 (0.12)	0.97
				CU A+ vs Dementia A+	-0.01 (0.18)	0.96
				MCI A+ vs Dementia A+	-0.02 (0.20)	0.94

© 2025 de Leeuw DM et al. *JAMA Network Open*.

eTable 3. Stage Specific Parameters for All Associations Between Cognitive Tests and CSF Biomarkers by Estimated Marginal Means of Linear Trends (Emtrends)

**Association**	**Stage**	**Emtrend estimate (SE)**	**P value**	**Stage contrast**	**Contrast estimate**	**P value**
**MMSE ~ Aß1-42/ Aß1-40 ratio**	Controls	1.74 (21.5)	0.94	Controls vs CU A+	-47.83 (52.0)	0.36
	CU A+	49.57 (47.2)	0.30	Controls vs MCI A+	-64.54 (62.4)	0.30
	MCI A+	66.28 (58.4)	0.26	Controls vs Dementia A+	-54.87 (47.7)	0.25
	Dementia A+	56.61 (42.5)	0.18	CU A+ vs MCI A+	-16.71 (74.9)	0.82
				CU A+ vs Dementia A+	-7.05 (62.7)	0.91
				MCI A+ vs Dementia A+	9.66 (72.4)	0.89
**Delayed recall ~ Aß1-42/ Aß1-40 ratio**	Controls	4.29 (21.6)	0.84	Controls vs CU A+	-98.0 (52.1)	0.06
	CU A+	102.29 (47.3)	**0.03**	Controls vs MCI A+	28.8 (63.5)	0.65
	MCI A+	-24.49 (59.6)	0.68	Controls vs Dementia A+	-32.7 (52.7)	0.53
	Dementia A+	37.03 (48.0)	0.44	CU A+ vs MCI A+	126.8 (75.9)	0.10
				CU A+ vs Dementia A+	65.3 (66.5)	0.33
**Association**	**Stage**	**Emtrend estimate (SE)**	**P value**	**Stage contrast**	**Contrast estimate**	**P value**

© 2025 de Leeuw DM et al. *JAMA Network Open*.

				MCI A+ vs Dementia A+	-61.5 (76.8)	0.42
**MMSE ~ pTau**	Controls	8.36e-3 (1.69e-2)	0.62	Controls vs CU A+	1.26e-2 (1.92e-2)	0.51
	CU A+	-4.27e-3 (9.95e-3)	0.67	Controls vs MCI A+	4.30e-2 (1.99e-2)	**0.03**
	MCI A+	-3.46e-2 (1.02e-2)	**<0.001**	Controls vs Dementia A+	1.73e-2 (1.78e-2)	0.33
	Dementia A+	-8.89e-3 (6.18e-3)	0.15	CU A+ vs MCI A+	3.03e-2 (1.43e-2)	**0.04**
				CU A+ vs Dementia A+	4.62e-3 (1.16e-2)	0.69
				MCI A+ vs Dementia A+	-2.57e-2 (1.19e-2)	**0.03**
**Delayed recall ~ pTau**	Controls	8.00e-3 (1.69e-2)	0.63	Controls vs CU A+	2.25e-2 (1.94e-2)	0.25
	CU A+	-1.45e-2 (1.00e-2)	0.15	Controls vs MCI A+	3.08e-2 (2.09e-2)	0.14
	MCI A+	-2.28e-2 (1.22e-2)	0.06	Controls vs Dementia A+	2.44e-2 (1.84e-2)	0.18
	Dementia A+	-1.64e-2 (7.67e-3)	**0.03**	CU A+ vs MCI A+	8.27e-3 (1.58e-2)	0.60
				CU A+ vs Dementia A+	1.89e-3 (1.26e-2)	0.88
**Association**	**Stage**	**Emtrend estimate (SE)**	**P value**	**Stage contrast**	**Contrast estimate**	**P value**
				MCI A+ vs Dementia A+	-6.38e-3 (1.43e-2)	0.66

© 2025 de Leeuw DM et al. *JAMA Network Open*.

**MMSE ~ tTau**	Controls	3.02e-4 (1.96e-3)	0.88	Controls vs CU A+	1.10e-3 (2.55e-3)	0.67
	CU A+	-7.94e-4 (1.70e-3)	0.64	Controls vs MCI A+	7.43e-3 (2.63e-3)	**0.005**
	MCI A+	-7.13e-3 (1.74e-3)	**<0.001**	Controls vs Dementia A+	2.16e-3 (2.20e-3)	0.33
	Dementia A+	-1.86e-3 (1.04e-3)	0.08	CU A+ vs MCI A+	6.34e-3 (2.43e-3)	**0.01**
				CU A+ vs Dementia A+	1.06e-3 (1.97e-3)	0.59
				MCI A+ vs Dementia A+	-5.28 (2.02e-3)	**0.01**
**Delayed recall ~ tTau**	Controls	-6.49e-4 (1.97e-3)	0.74	Controls vs CU A+	1.77e-3 (2.58e-3)	0.49
	CU A+	-2.42e-3 (1.73e-3)	0.16	Controls vs MCI A+	3.60e-3 (2.82e-3)	0.20
	MCI A+	-4.25e-3 (2.03e-3)	**0.04**	Controls vs Dementia A+	2.53e-3 (2.36e-3)	0.28
	Dementia A+	-3.18e-3 (1.35e-3)	**0.02**	CU A+ vs MCI A+	1.83e-3 (2.65e-3)	0.49
				CU A+ vs Dementia A+	7.54e-4 (2.17e-3)	0.73
**Association**	**Stage**	**Emtrend estimate (SE)**	**P value**	**Stage contrast**	**Contrast estimate**	**P value**
				MCI A+ vs Dementia A+	-1.07e-3 (2.42e-3)	0.66

© 2025 de Leeuw DM et al. *JAMA Network Open*.

eTable 4. Associations Between Cognitive tests and CSF Biomarkers Slopes and Intercepts

Note that models were only performed in groups that have shown changes over time in biomarkers and cognitive markers.

Association	Stage	Beta (SE) slope	P value	Beta (SE) intercept	P value
**MMSE ~ pTau**	MCI A+	-3.43 (1.45)	**0.02**	-0.37 (0.35)	0.25
**MMSE ~ tTau**	MCI A+	-4.00 (1.74)	**0.03**	-0.39 (0.44)	0.38
	Dementia A+	-0.23 (2.39)	0.92	1.74 (1.53)	0.26
**Delayed recall ~ Aß1-42/ Aß1-40 ratio**	CU A+	-0.49 (0.77)	0.53	0.31 (0.51)	0.55
**Delayed recall ~ pTau**	CU A+	-0.91 (0.77)	0.26	-0.51 (0.56)	0.36
	MCI A+	-0.89 (0.66)	0.18	0.09 (0.41)	0.83
**Delayed recall ~ tTau**	CU A+	-0.92 (0.91)	0.33	-0.70 (1.32)	0.60
	MCI A+	-0.76 (0.74)	0.31	0.46 (0.87)	0.59
	Dementia A+	-1.77 (1.39)	0.21	0.60 (1.05)	0.58

© 2025 de Leeuw DM et al. *JAMA Network Open*.
