# Supplementary material for: Cerebrospinal Fluid Amyloid and Tau Biomarker Changes Across the Alzheimer Disease Clinical Spectrum
Source: JAMA Netw Open. 2025 Jul 10;8(7):e2519919. doi: 10.1001/jamanetworkopen.2025.19919 (PMC12246881; doi:10.1001/jamanetworkopen.2025.19919)
Supplement: Supplement 2. — Data Sharing Statement [file jamanetwopen-e2519919-s002.pdf]

Data Sharing Statement

de Leeuw. Cerebrospinal Fluid Amyloid and Tau Biomarker Changes Across the Alzheimer Disease Clinical Spectrum. *JAMA Netw Open*. Published July 10, 2025. doi:10.1001/jamanetworkopen.2025.19919

Data
Data available: No
